# GEMBASSY: an EMBOSS associated software package for comprehensive genome analyses

**DOI:** 10.1186/1751-0473-8-17

**Published:** 2013-08-29

**Authors:** Hidetoshi Itaya, Kazuki Oshita, Kazuharu Arakawa, Masaru Tomita

**Affiliations:** 1 Institute for Advanced Biosciences, Keio University, 14-1, Baba town, Tsuruoka city, Yamagata Pref. 997-0035, Japan; 2 System Biology Program, Graduate School of Media and Governance, Keio University, 5322, Endo, Fujisawa city, Kanagawa Pref. 252-0882, Japan

## Abstract

The popular European Molecular Biology Open Software Suite (EMBOSS) currently contains over 400 tools used in various bioinformatics researches, equipped with sophisticated development frameworks for interoperability and tool discoverability as well as rich documentations and various user interfaces. In order to further strengthen EMBOSS in the fields of genomics, we here present a novel EMBOSS associated software (EMBASSY) package named GEMBASSY, which adds more than 50 analysis tools from the G-language Genome Analysis Environment and its Representational State Transfer (REST) and SOAP web services. GEMBASSY basically contains wrapper programs of G-language REST/SOAP web services to provide intuitive and easy access to various annotations within complete genome flatfiles, as well as tools for analyzing nucleic composition, calculating codon usage, and visualizing genomic information. For example, analysis methods such as for calculating distance between sequences by genomic signatures and for predicting gene expression levels from codon usage bias are effective in the interpretation of meta-genomic and meta-transcriptomic data. GEMBASSY tools can be used seamlessly with other EMBOSS tools and UNIX command line tools. The source code written in C is available from GitHub (https://github.com/celery-kotone/GEMBASSY/) and the distribution package is freely available from the GEMBASSY web site (http://www.g-language.org/gembassy/).

## Background

First released in the year 2000, the European Molecular Biology Open Software Suite (EMBOSS) [1] is a comprehensive package for sequence analyses consisting of over 400 tools and is one of the most popular bioinformatics software packages. EMBOSS is not merely a collection of software tools, but is equipped with rich documentation and development framework to achieve high level of software interoperability and discoverability based on the Ajax Command Definitions (ACD) metadata for the tools. EMBOSS, therefore, is an interoperable bioinformatics software platform which work seamlessly in concert with other UNIX command-line tools, and it can alternatively be accessed from graphical user interface JEMBOSS [2] or from web based interface EMBOSS Explorer [3]. Third-party development using the EMBOSS platform is called the EMBOSS associated software (EMBASSY), and we have previously developed an EMBASSY package named the Keio Bioinformatics Web Service (KBWS) [4], which complements EMBOSS tools with access to 42 major bioinformatics web services such as NCBI BLAST and WebLogo. As a further expansion of EMBOSS, we hereby present a novel EMBASSY package designated GEMBASSY. This package adds over 50 tools for genome analysis and gene-centric sequence manipulation from genome flatfiles, implemented using methods from the G-language Genome Analysis Environment (G-language GAE) [5-7]. G-language GAE contains over 100 programs for genome analysis where most of which are implemented with published algorithms, and each of the programs are implemented with a variety of options and produces graphical output where available. Analysis programs included in the G-language GAE such as for the identification of conserved sequence motifs with information theory [8,9], prediction of expression levels of genes from codon usage bias [10], visualization of GC skew [11] and prediction of replication origin and terminus [12,13], are effective in comparative study of bacterial genomes.

### Implementation

GEMBASSY is an EMBASSY package implemented on the EMBOSS platform and has 53 wrapper programs of the G-language web services [14]. The tools implemented in GEMBASSY are mainly consisted of whole genome analysis methods as listed in Table 1 (more detailed descriptions are given at the project web site: http://www.g-language.org/gembassy/). Programs are named according to the corresponding G-language GAE method name prefixed with the letter “g”. The software is written in C with EMBOSS Ajax libraries, gSOAP Toolkit [15], and additional source code files created from the G-language SOAP Web Service Description Language (WSDL) file (http://soap.g-language.org/g-language.wsdl) using the *wsdl2h* utility included in the gSOAP Toolkit. GEMBASSY tools basically handles file input on the client side, and runs the actual analysis on the G-language SOAP/REST server. By taking this client–server architecture, the users do not need to install and maintain the G-language GAE, and can take advantage of the computational resource provided by the web services. Results of the analysis is then fetched by the client program, and output in EMBOSS-compliant manner. In order to expedite the transfer of large genome flatfiles, GEMBASSY provides the “-accid” option to specify and send only the accession ID of the sequence instead of transferring the entire information. Graphs are plotted using the EMBOSS graph plotting library, based on the Comma-Separated Values (CSV) file produced by the G-language web server, so that the image can be shown on screen through the X11 Window System or written out to 13 image formats supported in EMBOSS. The G-language SOAP service has been updated since its release to support accession ID as input and to support the latest version of G-language GAE. The distribution package is available from the GEMBASSY web site (http://www.g-language.org/gembassy/) or from GitHub repository (https://github.com/celery-kotone/GEMBASSY) under the terms of GNU General Public License version 2. The EMBOSS Explorer interface is also available for use from the following URL (http://soap.g-language.org/gembassy/emboss_explorer/).

**Table 1:** Complete list of 53 tools implemented in GEMBASSY

***Category***	***Method name***	***Category***	***Method name***
***Data Retrieval***	gentrez		gbasezvalue
	genret		gconsensusz
***Display***	gcgr		gdeltagcskew
	gcircularmap		gdistincc
	gdnawalk		gfindoriter
	ggenomemap3		ggcsi
	gseq2png		ggcskew
***Nucleic Codon Usage***	gbui		ggcwin
	gcai		ggeneskew
	gcbi		ggenomicskew
	gcodoncompiler		gkmertable
	gdeltaenc		gldabias
	gdinuc		gnucleotideperiodicity
	genc		goligomercounter
	gew		goligomersearch
	gfop		gpalindrome
	gicdi		gqueryarm
	gp2		gquerystrand
	gphx		greporiter
	gsvalue		gscs
	gwvalue		gseqinfo
***Nucleic Composition***	gb1		gsignature
	gb2		gviewcds
	gbasecounter	***Nucleic Mutation***	gshuffleseq
	gbaseentropy	***Protein Composition***	gaminoinfo
	gbaseinformationcontent	***Protein Properties***	gaaui
	gbaserelativeentropy		

Implemented tools are mostly consisted of genome analysis methods for nucleotide composition, codon usage, and genome information visualization, which are mainly implemented with published algorithms. The letter “g” is prefixed to indicate the software as a tool included in the GEMBASSY package. Detailed documentations for each of the tools as well as full references are available at the GEMBASSY web site (http://www.g-language.org/gembassy/).

## Results and discussion

**Figure 1:**
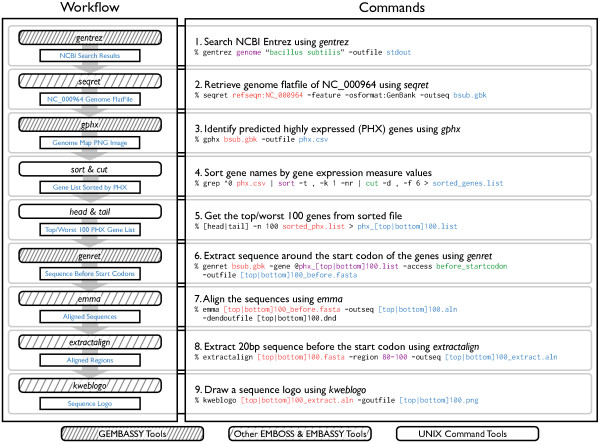
**Example analysis workflow with GEMBASSY.** The workflow first searches the NCBI Entrez Genome database for the term “*Bacillus subtilis*” (*gentrez*), retrieves the genome flatfile of the [RefSeq:NC_000964] entry (*entret*), and generates a sequence logo for sequences around start codons of top or worst 100 PHX (predicted highly expressed) genes (*gphx*, *emma*, *extractalign*, and *kweblogo*). This example seamlessly utilizes GEMBASSY, EMBOSS and regular UNIX commands.

An example analysis workflows with GEMBASSY, several other EMBOSS commands, EMBASSY tools including KBWS, and UNIX command-line tools is shown in Figure 1. The workflow of Figure 1 studies a bacterial genome and analyzes the conservation of Shine-Dalgarno (SD) sequence by comparing the nucleotide composition in top and bottom 100 highly and lowly expressed genes. SD sequences are located directly upstream of start codons, and are recognized by the ribosome for translation initiation [16].

**Figure 2:**
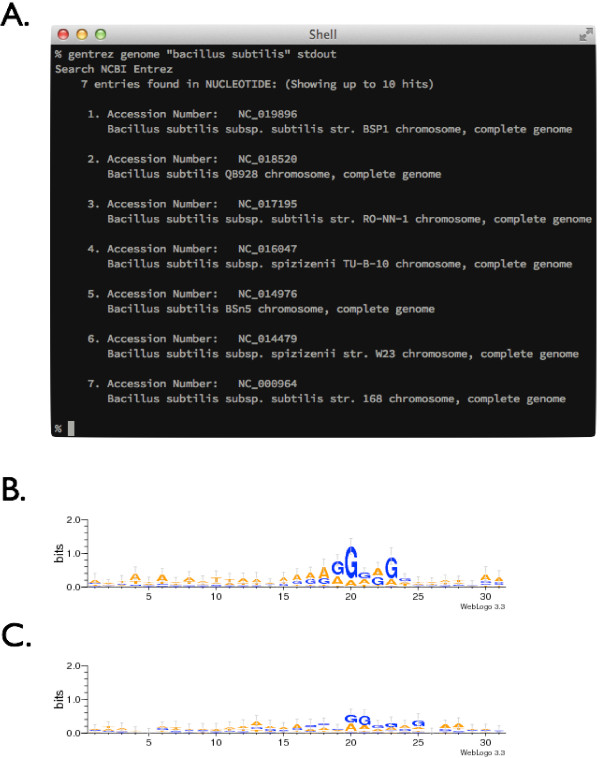
**Graphical output from the sample workflow. (A)** Result of keyword search in the NCBI Entrez Genome database with *gentrez* (result of Figure 1–1). **(B and C)** Sequence logos created with *kweblogo*, for top 100 **(B)** and worst 100 **(C)** PHX genes (Figure 1–9).

Here, the gene expression levels are predicted from synonymous codon usage bias based on the predicted highly expressed (PHX) index [17]. In this workflow, the accession ID of a genome of interest, which in this case is *Bacillus subtilis* [RefSeq:NC_000964], is searched in NCBI Entrez [18] using the *gentrez* utility of GEMBASSY (Figure 2-A). The flatfile of this genome is then retrieved using the *seqret* tool of EMBOSS. In order to study how SD sequence conservation is related to translation efficiency, the workflow next calculates the predicted gene expression level from the synonymous codon usage bias based on PHX (*gphx* tool). Using common UNIX commands *sort*, *cut*, *head*, and *tail*, top 100 highly expressed genes and bottom 100 lowly expressed genes are filtered as list of genes. Then, nucleotide sequences upstream of these genes are extracted from the genome flatfile using the *genret* utility. The *genret* tool is a versatile utility for the extraction of information in genome flatfiles, and users can easily retrieve feature information such as the gene product information and amino acid translation, as well as various nucleotide sequences such as those of the coding regions, exons, introns, or upstream and/or downstream regions. The *emma* utility is then used to align the sequences, and *extractalign* is used to extract the region of interest. Finally, these aligned upstream sequences are visualized using Sequence logos [19,20] to compare the conservation levels of SD sequence. As shown in Figures 2-B and 2-C, top 100 PHX genes (Figure 2-B) show slightly higher conservation of SD sequence than the worst 100 PHX genes (Figure 2-C), suggesting lower translation initiation efficiency in genes that are lowly expressed. Three other sample workflows along with the example above are available online at our web site (http://www.g-language.org/gembassy/), with detailed information of the workflows available at the GEMBASSY web site.

As exemplified in the workflow, GEMBASSY complements existing EMBOSS/EMBASSY tools for the manipulation of genome flatfiles and adds numerous analysis tools suited for genome-level studies available in the G-language GAE. By making the tools available as an EMBASSY package based on EMBOSS framework, the users can use the same documentation (*tfm*) and discovery tools (*wossname*) of EMBOSS, and can take advantage of the familiar user interface that they are accustomed to.

## Abbreviations

ACD: Ajax command definition; CSV: Comma-separated values; EMBOSS: European Molecular Biology Open Software Suite; PHX: Predicted highly expressed; REST: Representational state transfer; SD: Shine-Dalgarno; WSDL: Web service description language.

## Competing interests

The authors declare that they have no competing interests.

## Authors’ contributions

HI developed the software, KO updated the G-language SOAP service, KO and KA conceived of the project, and HI, KO, and KA designed the software and drafted the manuscript. MT supervised the project. All authors have read and approved the final manuscript.
